# Unoccupied aerial systems discovered overlooked loci capturing the variation of entire growing period in maize

**DOI:** 10.1002/tpg2.20102

**Published:** 2021-05-19

**Authors:** Alper Adak, Seth C. Murray, Steven L. Anderson, Sorin C. Popescu, Lonesome Malambo, M. Cinta Romay, Natalia de Leon

**Affiliations:** ^1^ Dept. of Soil and Crop Sciences Texas A&M Univ. College Station TX 77843‐2474 USA; ^2^ Dept. of Environmental Hort., Institute of Food and Agricultural Sciences, Mid‐Florida Research and Education Center University of Florida Apopka FL USA; ^3^ Dept. of Ecosystem Science and Management Texas A&M Univ. College Station TX 77843‐2120 USA; ^4^ Institute for Genomic Diversity Cornell University Ithaca NY USA; ^5^ Department of Agronomy University of Wisconsin 1575 Linden Drive Madison WI 53706 USA

## Abstract

Traditional phenotyping methods, coupled with genetic mapping in segregating populations, have identified loci governing complex traits in many crops. Unoccupied aerial systems (UAS)‐based phenotyping has helped to reveal a more novel and dynamic relationship between time‐specific associated loci with complex traits previously unable to be evaluated. Over 1,500 maize (*Zea mays* L.) hybrid row plots containing 280 different replicated maize hybrids from the Genomes to Fields (G2F) project were evaluated agronomically and using UAS in 2017. Weekly UAS flights captured variation in plant heights during the growing season under three different management conditions each year: optimal planting with irrigation (G2FI), optimal dryland planting without irrigation (G2FD), and a stressed late planting (G2LA). Plant height of different flights were ranked based on importance for yield using a random forest (RF) algorithm. Plant heights captured by early flights in G2FI trials had higher importance (based on Gini scores) for predicting maize grain yield (GY) but also higher accuracies in genomic predictions which fluctuated for G2FD (−0.06∼0.73), G2FI (0.33∼0.76), and G2LA (0.26∼0.78) trials. A genome‐wide association analysis discovered 52 significant single nucleotide polymorphisms (SNPs), seven were found consistently in more than one flights or trial; 45 were flight or trial specific. Total cumulative marker effects for each chromosome's contributions to plant height also changed depending on flight. Using UAS phenotyping, this study showed that many candidate genes putatively play a role in the regulation of plant architecture even in relatively early stages of maize growth and development.

## Core Ideas

- Temporal plant heights can predict maize hybrids via machine learning regression.
- Early plant heights were more important in yield prediction under optimal conditions.
- Segregating loci affecting maize plant height varied temporally and were determined by stress.
- Genomic predictions varied depending on plant heights belonging to different flight dates.
- Genomic prediction accuracy can be increased with using temporal phenotype data.

**Abbreviations**
BLUP: best linear unbiased prediction
DAP: days after planting
DTA: days to anthesis
DTS: days to silking
G×E: genotype × environment
G2F: Genomes to Fields
G2FD: optimal dryland planting without irrigation
G2FI: optimal planting with irrigation
G2LA: stressed late planting
GAPIT: genome association and prediction integrated tool
GBS: genotyping‐by‐sequencing
GDD: growing degree days
GY: grain yield
HTP: high‐throughput phenotyping
LD: linkage disequilibrium
LM: linear model
QTL: quantitative trait loci
RF: random forest
*r*_gpa_
: genomic prediction accuracies
SNP: single nucleotide polymorphism
UAS: unoccupied aerial systems
UAS_GEBVs_
: genetically estimated breeding value for each flight
UAS_PEBV_
: phenotypically estimated breeding value for each flight

## 1 INTRODUCTION

Quantitative variation of complex traits in maize (*Zea mays* L.) have been challenging to dissect since they show strong environmental interactions and are generally inconsistent between populations and different screening environments (Beavis et al., 1991; Koester et al., 1993; Peiffer et al., 2014; Sari‐Gorla et al., 1999; Veldboom & Lee, 1996; Wang et al., 2006). Plant height, traditionally measured terminally at the end of the growing season with a ruler, is a prime example of a quantitative, complex trait; it is relatively easy to measure across many plots and it has high repeatability and heritability (Anderson et al., 2018,2019, 2020; Mahan et al., 2018; Peiffer et al., 2014; Rood & Major, 1981; Veldboom & Lee, 1996). Genetic mapping and theory suggest an omnigenic model supported by the genetically polygenic inheritances observed and the variable contributions of pedigree as a source of variation, consistent with a large number of loci with minor effects governing these traits (Boyle et al., 2017; Mackay, 2001; Peiffer et al., 2014; Wallace et al., 2016; Wang et al., 2006). Recently, emerging high‐throughput phenotyping (HTP) tools have demonstrated that complicated phenotypically dynamic growth patterns are occurring (Anderson et al., 2020; Miao et al., 2020; Pauli et al., 2016; Wang et al., 2019; Wu & Lin, 2006) and that the importance of various loci change temporally throughout plant growth and development (Furbank & Tester, 2011).

Using ruler‐based terminal plant height, a limited number of genes, well‐known to play a role in hormone synthesis and signaling pathways in maize, have been found. These larger‐effect plant height genes are involved in processes such as regulating gibberellin signaling and biosynthesis (Bensen et al., 1995; Lawit et al., 2010; Winkler & Helentjaris, 1995), hindering of polar movement of auxin transport (Multani et al., 2003), and brassinosteroid synthesis (Hartwig et al., 2011; Makarevitch et al., 2012). Disruption of such hormone synthesis and signaling genes can cause significant reductions in plant heights. Although application to breeding have remained limited because of an antagonistic effect on yield (Bensen et al., 1995; Thornsberry et al., 2001; Winkler & Helentjaris, 1995), genetic variants at large‐effect hormone loci may have been fixed or found to not segregate widely as a consequence of directional selection over time (Peiffer et al., 2014). As a phenotype, terminal plant height can also be a strong predictor of yield in some environments even in elite commercial hybrids (Farfan et al., 2013). It has been observed that early plant height measures (i.e., in seedling, jointing, and flowering growth stages; V0–VT) may provide novel insights into maize yield (Machado et al., 2002) and have the potential to predict yield at earlier time points (Anderson et al., 2019; Miao et al., 2020; Yin et al., 2011a,b; Zhang et al., 2017). It is likely that many quantitative trait loci (QTL) studies have been limited in explaining erratic plant height variation because they relied on end‐of‐season terminal height phenotyping data that did not monitor plant architecture and environmental stresses throughout plant growth (Su et al., 2019; Wang et al., 2010). Partitioning the genetic component from the total variation at each growth stage in a timely and repeated manner might further help to explain the hidden heritability issue in genetic dissection (Gibson, 2010).

**Core Ideas**

- Temporal plant heights can predict maize hybrids via machine learning regression.
- Early plant heights were more important in yield prediction under optimal conditions.
- Segregating loci affecting maize plant height varied temporally and were determined by stress.
- Genomic predictions varied depending on plant heights belonging to different flight dates.
- Genomic prediction accuracy can be increased with using temporal phenotype data.

Complex traits are orchestrated by the interplay of many genes, some constitutively expressed during all growth periods, while others are expressed at specific time periods (Feldman et al., 2017; Tessmer et al., 2013; Bac‐Molenaar et al., 2015; Schmid et al., 2005; Sun & Wu, 2015; Li & Sillanpää, 2015). High‐throughput phenotyping can measure the physical characteristics of plants in a temporal manner and has previously been used to discover temporal QTL under field conditions for rice (*Oryza sativa* L.) (Tanger et al., 2017; Yang et al., 2014), triticale (×*Triticosecale* spp.) (Würschum et al., 2014), cotton (*Gossypium* spp.) (Pauli et al., 2016), barley (*Hordeum vulgare* L.) (Neumann et al., 2017), and wheat (*Triticum aestivum* L.) (Lyra et al., 2020). In order to capture temporal gene–trait associations in maize, two studies have used automated greenhouse‐based HTP platforms (Junker et al., 2015) under environmentally controlled conditions (Zhang et al., 2017; Muraya et al., 2017), while two others used unoccupied aerials system (UAS)‐based HTP platforms under field conditions (Anderson et al., 2020; Wang et al., 2019) but only for inbred lines and not hybrids, which are what farmers grow.

Repeated data collection of maize plant height by UAS has already shown dynamic correlations with yield at different time points in a segregating population of hybrid lines (Anderson et al., 2019). Within the scope of the Genomes to Fields (G2F) project, UAS HTP data captured the temporal variations of growth patterns in diverse hybrids as well as other agronomic traits (i.e., flowering times and yield) under three different environmental management conditions (optimal planting with irrigation [G2FI], optimal planting without irrigation [G2FD], and late planting with irrigation [G2LA]) during 2017. In breeding programs, a predictive model that can quickly identify the highest yielding cultivars to advance before or without harvest yield data in larger nurseries would facilitate faster decisions, saving time and resources and possibly shortening the breeding cycle.

The objectives of this study were to (a) use temporal plant heights of the maize hybrids belonging to 22 flight dates (corresponding to 60 time points for three trials) as predictor variables in order to predict maize yield via a random forest (RF) algorithm; (b) dissect the underlying temporal QTL associated with the variation captured by each flight via a genome‐wide association study; and (c) estimate and compare the temporal genomic predictions for plant height of each flight for each trial.

## 2 MATERIALS AND METHODS

### 2.1 Genetic materials, experimental conditions, and high‐throughput phenotyping

The G2F project is an umbrella initiative involving collaborators from a variety of disciplines aiming to perform high‐throughput genotyping and phenotyping to understand gene × gene (G×G) and genotype × environment (G×E) interactions in maize (https://www.genomes2fields.org). Under this project, a HTP platform via UAS was used to capture plant growth during various development stages over three management conditions per year in College Station, TX. In 2017, 280 hybrids were planted on 3 March under G2FI, while 230 hybrids of the same set were planted on 3 March under G2FD, and on 6 April for G2LA in College Station, Texas. To visualize the heat stress to which the G2LA has been exposed, cumulative growing degree days (GDD, °C) and photoperiod (h) were illustrated for the first 150‐d period following optimal (G2LE and G2FD) and delayed (G2LA) planting times (Supplemental Figure S1). Growing degree days were calculated per day by subtracting the average of the daily maximum and minimum temperature from the base temperature (10 °C). If GDD was less than zero for any given day, it was set to zero. All hybrids in each trial were grown based on a randomized complete‐block design with two replications. In each replication of each trial, all hybrids were grown as two adjacent row plots (7.62‐m row plot length and 0.76‐m row plot spacing between all rows) and grain yield (GY) was collected from two adjacent row plots per hybrid with a plot combine harvester. Days to anthesis (DTA) and silking (DTS) were recorded for each hybrid as the number of days from the time of planting until at least 50% of each hybrid showed anthesis and silking emergence in the two row plots. Agronomic field data and weather data is available for 2017 (https://doi.org/10.25739/w560‐2114) (McFarland et al., 2020).

Preliminary studies of image processing and plant height extraction from HTP platform for the three trials of 2017 were reported previously by Anderson et al. (2019). First, point clouds were clipped based on the trial boundaries of G2FI, G2FD, and G2LA. Then noise points, which are located at the far bottom and top of the point clouds were illustrated by using the lateral view function in CloudCompare v2.10 (Girardeau‐Montaut, 2016). These noise points can cause big fluctuations in plant height data extraction, so they were manually selected using segment tool function of CloudCompare v2.10 and removed from point cloud data. Executable functions of LAStools (Isenburg, 2015; rapidlasso, 2017) and FUSION/LDV (McGaughey, 2016) software were transported in R. A custom batch script was developed with several steps to extract the plot‐based plant height data. R/UAStools package was used to create the polygons (ESRI shapefile) for each two adjacent row plots in each trial using the unique plot IDs (Anderson & Murray et al., 2020); two adjacent row plots‐based polygon construction was also illustrated in plotshpcreate.R (https://github.com/andersst91/UAStools/wiki/plotshpcreate.R). A brief overview of the plant height extraction steps of custom batch script from point cloud data in R was described as follows: (a) points of clipped point cloud data of each trial were first sorted using the function of lasssort.exe of LAStools software; (b) the noisy points that were so close to the canopy structure of plants in row plots were removed using the function of lasnoise.exe of LAStools before constructing the digital surface model; (c) ground points were identified using the hierarchical robust interpolation algorithm (Kraus & Pfeifer, 1998) using the function of GroundFilter.exe of FUSION software; (d) maximum points from the ground points were determined using the function of lasthin.exe of LAStools software; and finally (e) a digital terrain model was generated based on the maximum points from the previous steps using the function of GridSurfaceCreate.exe of FUSION software. Next, canopy surface models generated from the digital terrain model (output of step [e]) were extracted from the digital surface model (the output of step [b]). Row plots in canopy surface models then were clipped based on the previously generated ESRI shapefile using the function of PolyClipData.exe of FUSION software. As a final step, plant heights were extracted for each clipped cloud points of each row plot as a percentile metrics using the function of CloudMetrics.exe of FUSION software. This study only used 99‐percentile‐based plant heights (Malambo et al., 2018). All flight dates were converted into days after planting (DAP) and specified DAP in the text, figures, and tables in this study.

The point cloud data (and all raw data) is available on Cyverse (Murray et al., 2019). In total, 21, 20, and 19 flights were used for G2FI, G2FD, and G2LA trials, respectively. For plant height, obvious outliers (>3‐m tall) were removed from the phenotypic data. Missing phenotypes (two hybrids at 36 and 39 DAP in G2LA) were imputed using missForest package in R.

### 2.2 Statistical models and RF application to HTP data

In order to calculate phenotypically estimated breeding values for each flight (UAS_PEBVs_), a full random model was fit using standard least squares (restricted maximum likelihood method) in JMP version 15 Pro (SAS Institute Inc.). Best linear unbiased predictions (BLUPs) of hybrids along with all random effects of variance components were estimated based on Equation 1:

(1) $$\begin{array}{ccc} y_{grii} & = & \;\mu +Genotype_{g}+\;Replation_{r}+\;Range_{i}+Row_{j}\\ & & +\;\varepsilon_{grij} \end{array}$$

where **y** is the response vector of plant heights belonging to each flight time, μ is grand mean, **g** is the vector of genetic effects of the hybrids [$g\;∼\;NID(0,I\sigma_{g}^{2})$], **r**, **i**, and **j** are replication $\left[r\;∼\;NID(0,I\sigma_{r}^{2})\right]$, field range $\left[i\;∼\;NID(0,I\sigma_{i}^{2})\right]$ and field row $\left[j\;∼\;NID(0,I\sigma_{j}^{2})\right]$ effects, respectively, accounting for spatial effects in the randomized complete block design; **ε** is the vector of error $\left[\varepsilon ∼NID(0,I\sigma_{\varepsilon }^{2})\right]$. **Genotype**, **replication**, **range**, and **row** are the incidence matrices of the variance components in the model.

Repeatability was estimated as follows (Equation 2):

(2) $$R\;=\frac{\sigma_{g}^{2}}{\sigma_{g}^{2}+\frac{\sigma_{\varepsilon }^{2}}{no.\;of\;reps}}\;$$

Pearson correlation coefficients were also calculated using UAS_PEBVs_ belonging to each flight of each trial separately to show the correlations among plant heights of different time points.

The UAS_PEBVs_ belonging to each flight of each trial were used as predictors to predict continuous yield. Both linear model (LM) and random forest (RF) regressions were applied to each trial separately. The training and test populations were set as 70 and 30%, respectively. The Caret package was used to implement two regression models in R by setting the method=lm for LM and method=rf for RF models. *K*‐fold cross‐validation was implemented by using the trainControl function of Caret package for both models. To conduct the 10‐fold with three replications cross‐validation, method, number, and repeats were set repeatedcv, 10 and 3, respectively. Root mean square error, mean absolute error, and *R* ^2^ were used to assess models for each trial. To tune the parameters of RF, tunegrid was used to find best number of predictors sampled for splitting at each node (*m*_try_) and number of trees grown (ntree) based on lowest RMSE. To illustrate the importance of the UAS_PEBVs_ belonging to each flight, varImp function was used after RF model, where higher varImp scores indicate higher importance of the explanatory variable (flight dates) in prediction the yield. To show the relationships between each predictor (flight dates) and outcome (yield) in the RF model, partial dependence plots were generated by using the pdp package in R (Friedman, 2001).

To assess prediction skill of the LM and RF models, predict function of Caret caret::predict() was used to predict yield of test data set using 1,000 bootstraps. Correlations between actual yield of test data and predicted yield of test data were calculated for LM (*r*_lm_) and RF (*r*_rf_) models at each bootstrap. The Wilcoxon signed rank test was then applied to each trial to compare correlation results of the LM and RF models.

### 2.3 SNP discovery and association mapping

More than 1,500 inbred lines with a total of 955,690 SNPs (GBS v2.7) were produced using Illumina Hi‐seq 2000/2500 at the Institute for Genomic Diversity, Cornell University, Ithaca, NY, USA (Glaubitz et al., 2014). The imputed ZeaGBSv2.7 data is also available through CyVerse with AGPv4 physical coordinates (McFarland et al., 2020; www.panzea.org; Elshire et al., 2011). Genotyping‐by‐sequencing (GBS) data of 158, 118, and 118 hybrids from 2017 was synthesized from sequence information of the available parental inbreds for the G2FI, G2FD, and G2LA trials, respectively. Tassel 5 software was used to call polymorphic markers (Bradbury et al., 2007). First, markers were set as missing if the either or both inbreds of hybrids used in this study were heterozygous. Second, sequence information of each hybrid was generated using the Create_Hybrid_Genotype function in Tassel 5. Third, markers with missing data (>10%) and minor allele frequency of <1% were filtered out. Missing markers were imputed using SNP.impute=major code in GAPIT (Genome Association and Prediction Integrated Tool) package in R (Lipka et al., 2012). Finally, 153,252 markers remained.

Plant heights of hybrids, using the BLUPs in Equation 1, captured by each flight were associated with filtered GBS data in the three trials individually, implementing the fixed and random model circulating probability unification (Liu et al., 2016) with principal components in GAPIT (Lipka et al., 2012). In GAPIT, the Model.selection=TRUE code was used to determine the optimal number of principal components based on Bayesian information criterion. The first three principal components were used in genome‐wide association study (Supplemental Figure S2). To calculate the explained variation by the SNPs with a higher score than conservative Bonferroni correction [−log_10_ (*p*) > 6.5; 0.01/(no. of markers)] , a Random.model=TRUE code was used in GAPIT function. In addition, a false‐positive discovery rate was set [−log_10_ (*p*) > 5] to determine the same SNPs (if any), which were discovered with Bonferonni correction in one flight, were important in any other different flight. MaizeGBD (http://www.maizegdb.org/) was used to look up SNPs positions for determining the candidate genes in those regions using AGPv4 physical coordinates of B73. The Gramene database (http://www.gramene.org) was used to determine the functions of candidate genes. Linkage disequilibrium (LD) was examined for colocalized SNPs that were discovered in more than one flight or trial using *R* ^2^ (>0.8) in LD heatmap package in R (Shin et al., 2006).

### 2.4 Genomic prediction of flights

Genome‐wide prediction was applied to each flight of each trial to calculate genetic estimated breeding values (UAS_GEBVs_) using ridge regression best linear unbiased prediction package in R (Endelman, 2011). Training and test data sets were arranged at 70 and 30%, respectively. Equation 3 was used to estimate GEBVs:

(3) y=1μ+Zβ+ε

where **y** is the vector of observations as BLUPs of plant heights of flights, μ is the overall mean, **Z** is the marker matrix,  **β** is the marker effects matrix. and **ε** is the residual effects vector with the assumptions of $\beta ∼NID(0,I\sigma_{\beta }^{2})$ and $\varepsilon ∼NID(0,I\sigma_{\varepsilon }^{2})$.

Prediction accuracies for genomic predictions (*r*_gpa_) were obtained for each flight using correlations between UAS_PEBVs_ and UAS_GEBVs_ of test data set, which were calculated by using cross‐validation with 500 iterations.

## 3 RESULTS AND DISCUSSION

### 3.1 Variance components, repeatability, and breeding value estimations of hybrids using HTP data

**FIGURE 1:**
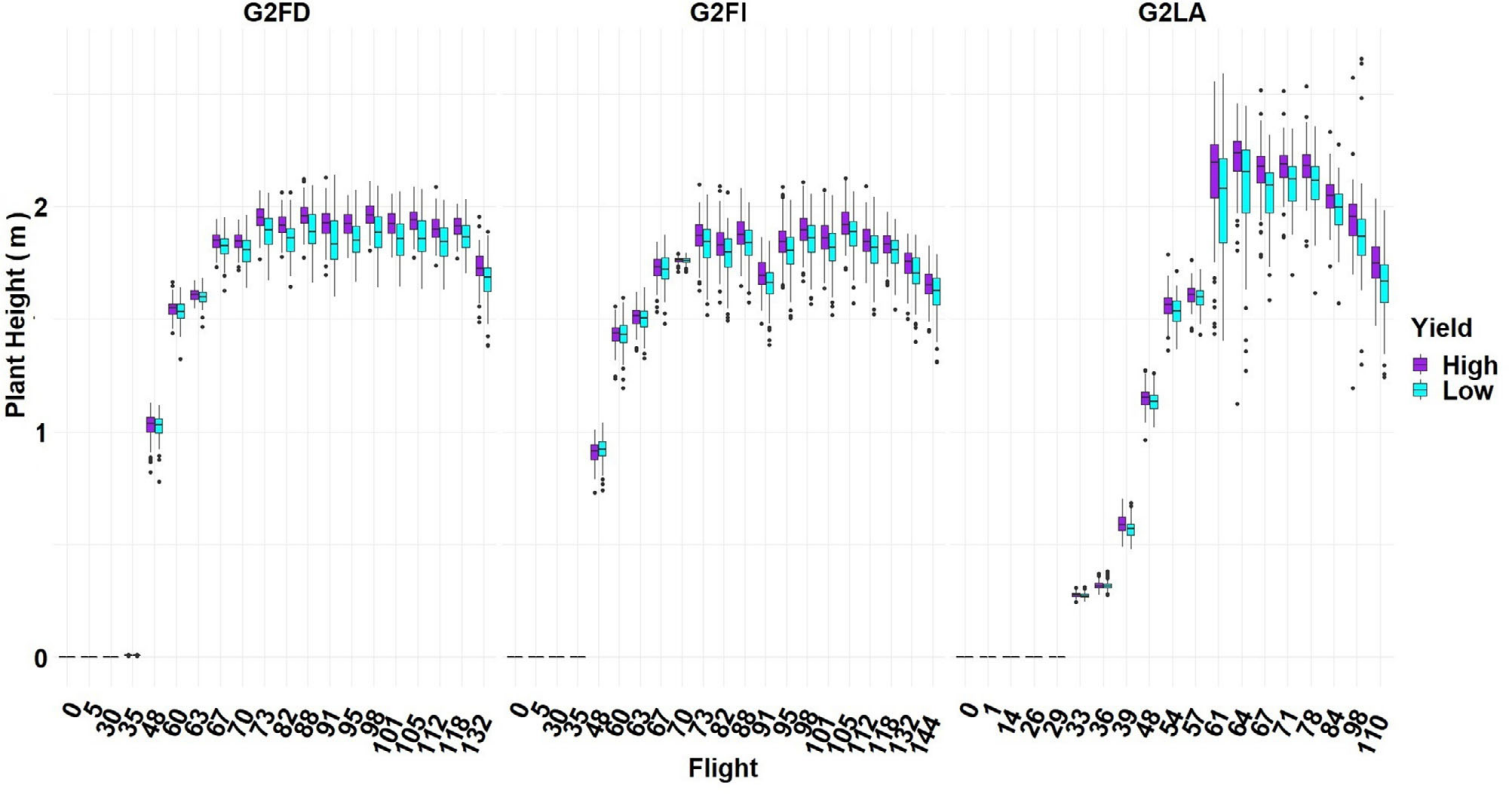
Illustrations of categorizing the plant heights of each flight of the hybrids grown in 2017 according to the high and the low yield values. G2FI, G2FD, and G2LA trial represent the optimal planting time with irrigation, optimal planting time without irrigation (dryland), and delayed planting time (late) with irrigation, respectively. *X* axes represents the flights as days after planting. *Y* axis represent the best linear unbiased predictions of plant heights (as meter unit) of both hybrids’ categories; high yield and low yield hybrid categories were represented by purple and cyan color box plots, respectively, for each flight date. Every hybrid was categorized as low‐ or high‐yielding hybrids if they had lower and higher yield value than average yield value of regarding trials

Using plot‐based plant height extraction from the 2017 G2F project in College Station, TX, BLUPs of plant heights belonging to maize hybrids for each flight date (UAS_PEBVs_) were estimated using Equation 1. The UAS_PEBVs_ were categorized into two groups: low and high yielding hybrids using the average yield threshold of each trial. As expected from previous work, taller hybrids, especially at flights after flowering, were relatively higher yielding than hybrids that were shorter (Figure 1).

Late plantings (G2LA) being taller is consistent with our normal observations in our breeding program over the years. Our best reason for this result is that hybrids were exposed to longer day length with higher heat accumulation (as illustrated in Supplemental Figure S1) when planted late (e.g., G2LA in this research). We do not know the entire mechanism but speculate it has to do with a combination of photoperiod (our plantings in Texas have been the only G2F location where plants flower as the days are getting longer, the later planting flowers closer to the longest day of the year) and with temperature (more rapid accumulations of GDD). These environmental conditions caused by late planting may cause the maize hybrids to produce more photosynthetic activity resulting in taller plant height and biomass but less yield.

**FIGURE 2:**
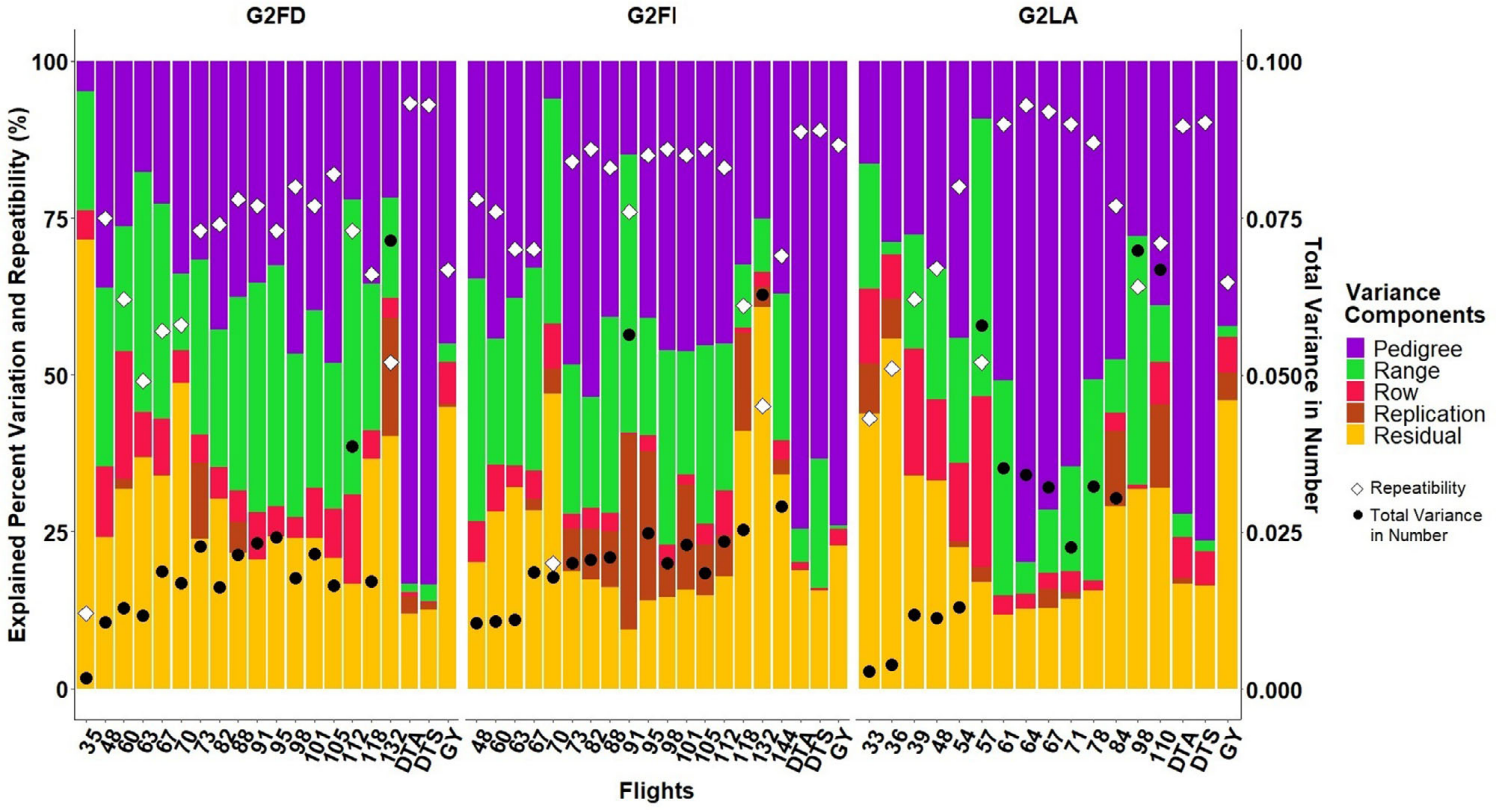
Stacked bar graph showed the explained percentage variations by each component calculated by Eq. 1 with repeatability and total variance (as number) for each trial. *X* axis is the flight as days after planting (DAP). Left *y* axis was scaled as percentage to show the repeatability values (white diamond) and percent variations by components. Right y axis was scaled as number to show the total variation explained as number (black round). DTA, DTS, and GY are the abbreviations of days to anthesis, silking (days), and plot‐based grain yield (t ha^−1^), respectively. Total variance of DTA, DTS, and GY in number are 5.67, 5.80, and 3.48 for G2FD; 4.22, 3.99, and 2.24 for G2FI; and 4.59, 4.32, and 1.49 for G2LA. G2FI, G2FD, and G2LA trials represent the optimal planting time with irrigation, optimal planting time without irrigation, and delayed planting time with irrigation respectively

The UAS_PEBVs_ variation explained by pedigree fluctuated between 5 to 80% of total variation explained by the model. Repeatability ranged from 12 to 93% across flights and trials (Figure 2). The hybrids with continuous taller plant heights across flights resulted in higher yield (Figure 1) even though repeatability results were not consistently estimated for plant heights through the flights. In addition, correlation coefficients among the plant heights (UAS_PEBVs_) belonging to later flights (after flowering times) had higher and more consistent correlation coefficients than the correlation coefficients of the plant heights (UAS_PEBVs_) belonging to earlier flights (before flowering times) (Supplemental Figure S3)

It is likely that differences in repeatability for each flight were related to the image and stitching quality of each flight (Anderson et al., 2019; Malambo et al., 2018) as well as weed pressures especially in earliest flights (e.g., 35 DAS in G2FD). It is also likely that plant height at any point in time was impacted by the activity of many genes and interplays between genes in response to changing environmental conditions during various growth periods (Veldboom & Lee, 1996; Messmer et al., 2009; Sibov et al., 2003; Anderson et al., 2020; Dijak et al., 1999; Han et al., 2018). Since UAS allowed temporal variation of plant height to be estimated here, this variation can be used in dissecting underlying genetic mechanisms such as discovering time‐specific and colocalized genes in association mapping.

### 3.2 Random forest algorithm for determining the importance ranks of flights

**FIGURE 3:**
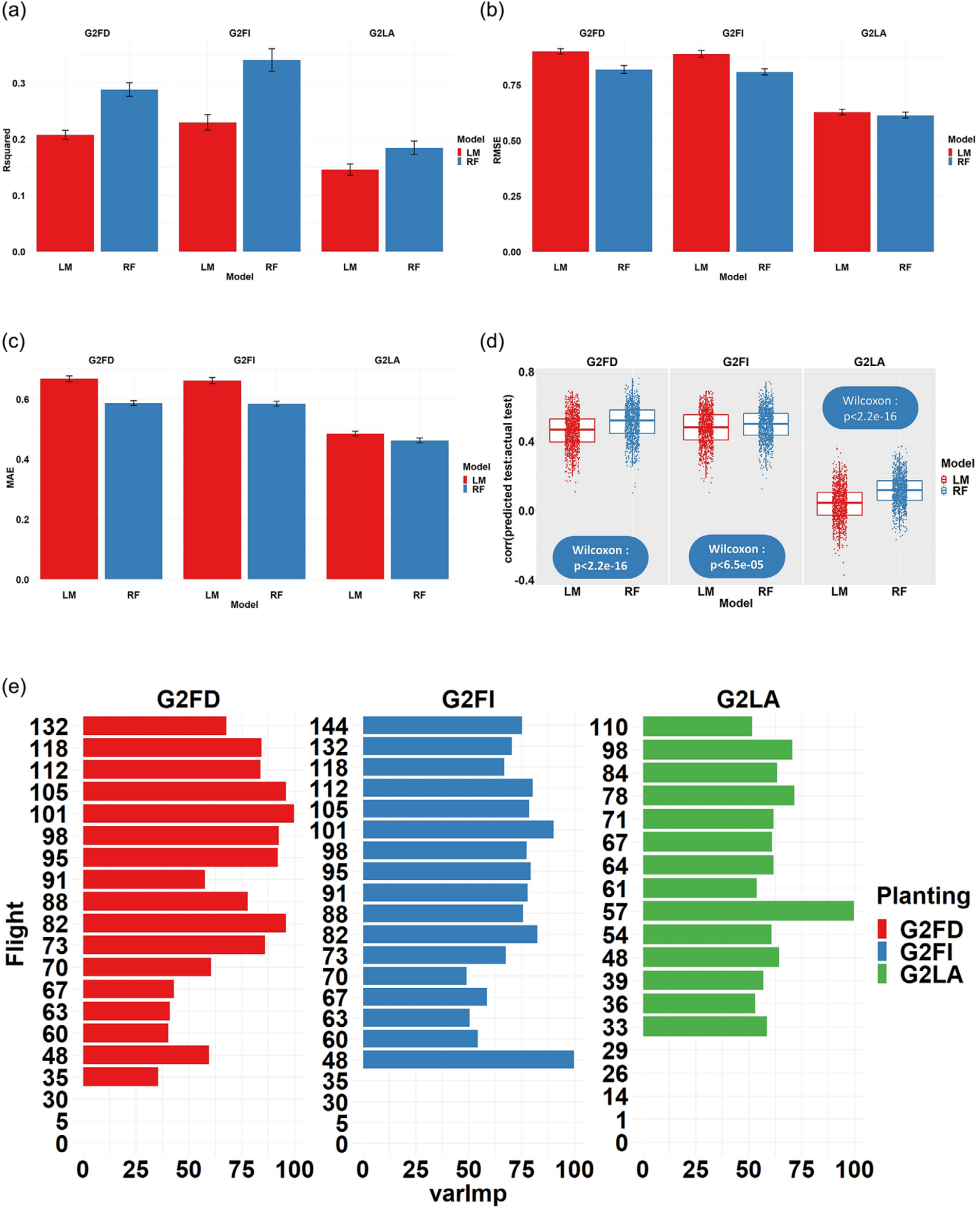
Model evaluations and variable importance scores. (a) The *R* ^2^ of linear (LM) and random forest (RF) model, higher is better. (b) Root mean square error (RMSE) of each model in each trial, lower is better. (c) Mean absolute error (MAE) of each model in each trial, lower is better. (d) Correlation results between predicted yield and actual yield of test data obtained by 1,000 bootstrap belonging to each model in each trial. Wilcoxon sign rank test results showed the comparison of correlations belonging to both analysis models in each trial. (e) Variable importance scores (varImp) show the variable importance scores of the predictors (flight dates) where higher varImp score indicates more importance variable in prediction the yield. G2FI, G2FD and G2LA trial represent the optimal planting time with irrigation, optimal planting time without irrigation, and delayed planting time with irrigation, respectively

**FIGURE 4:**
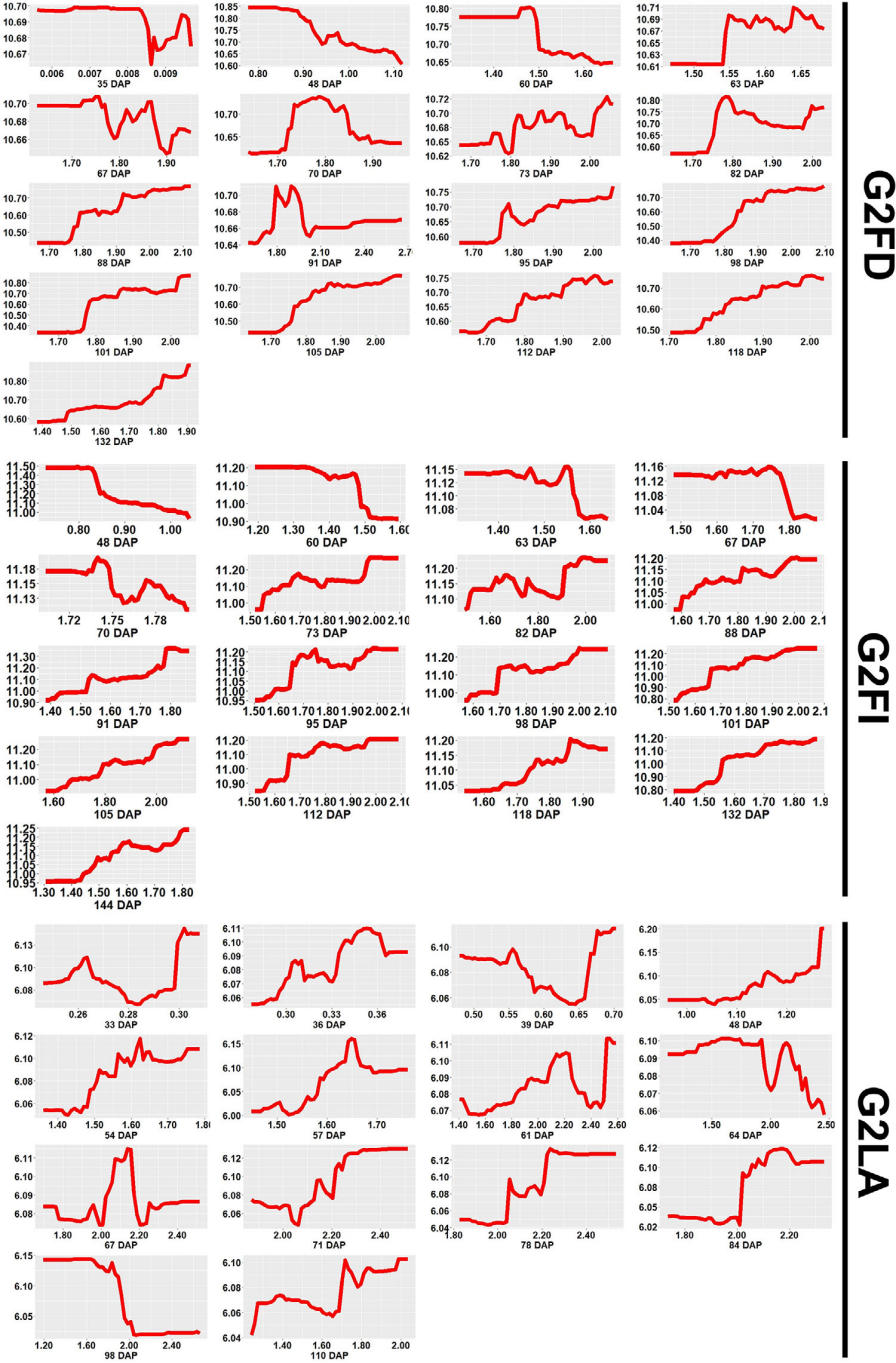
The partial dependence plots for each predictor (flight dates as days after planting; DAP) in each trial. *Y* axis of each plot shows the predicted continuous yield (t ha^−1^), while *x* axes are the predictor values that are plant heights of each flight dates. Red lines show the relationships between each predictor and predicted yield values. G2FI, G2FD, and G2LA trial represent the optimal planting time with irrigation, optimal planting time without irrigation, and delayed planting time with irrigation, respectively

Plant heights captured by various flights from the three different trials were used as predictors to predict GY using the LM and RF algorithm. The RF model was consistently found to perform better than the LM model in predicting yield in each trial based on *R* ^2^, RMSE, and mean absolute error (Figure 3). Prediction accuracies of the RF model (*r*_rf_) were found to be greater than prediction accuracies of LM model (*r*_lm_) in each trial (Figure 3), indicating that the RF model works slightly better in predicting GY using temporal plant heights. Variable importance scores (varImp) were used to determine variable (flight dates) importance (Figure 3). Results of varImp scores showed that different flight dates were most important across different trials; earlier flights (e.g., 48 DAP in G2FI and 52 DAP in G2LA) and later flights (e.g., 82 and 101 DAP in G2FD and 78 and 98 DAP in G2LA) had the highest scores (Figure 3). The partial dependence plots are a functional illustration for evaluating the relationship between each explanatory variable (flight dates) and the response variable (yield); the relationships might be linear, parabolic, or something even more complex (Friedman, 2001). The partial dependence plot shows the changing predicted yield values based on the plant heights at a particular time point; illustrations of this change are critical to understand the relationships between plant heights at different flight dates and yield. Partial dependence plots of every flight date in each trial were illustrated (Figure 4). For instance, the most important variable (based on varImp scores) was found to be 101 DAP in G2FD, 48 DAP in G2FI, and 57 DAP in G2LA (Figure 3). Plant height between 2.00 and 2.05 m at 101 DAP in G2FD, plant height between 0.75 and 0.80 m at 48 DAP in G2FI, and plant height between 1.60 and 1.70 m at 57 DAP in G2LA were found to be desired plant heights that attached to maize hybrids with higher yield values (Figure 4). There were additional plant height thresholds belonging to each flight times that can be used as selection criteria for higher yielding hybrids, considering the relationships between plant height at particular time points and yield (Figure 4). The majority of plant heights belonging to the flight dates were found to have nearly linear relationships with predicted yield values except for first three flights in G2FD and first four flights in G2FI (Figure 4). However, it is noteworthy to mention that linear relationships between plant heights of majority flights in G2FI is more obvious than G2FD and G2LA, indicating that stress factors in G2FD and G2LA caused the trend of linear relationships between plant heights and higher GY to change. In addition, taller plant height in earlier flight dates (33, 36, 39, and 48 DAP in G2LA) were better indicators of higher yield in G2LA than G2FI and G2FD (Figure 4). Different contributions of plant heights belonging to different growth periods and environments in predicting GY suggests that discovering genes controlling the plant height variation at different growth periods will provide supportive genetic information to manipulate yield in maize.

In general, the slightly convex shape distribution in varImp scores of flight dates in G2FD and G2FI trial (Figure 3) showed that 82–112 DAP in G2FI and 73–88 and 95–118 DAP in G2FD contributed to higher GY than other flight dates. The same flight dates varied in terms of varImp scores in G2FD and G2FI, especially later flight dates were found to have grater varImp scores in G2FD than G2FI. This is interesting because G2FI and G2FD trials were planted and flown at the same time; however, they were managed differently for optimal and dryland conditions, respectively. This suggests that plant height belonging to different growth periods of these management conditions affected GY differently; while this has long been hypothesized (Haghighattalab et al., 2017; Sun et al., 2019) and shown for a few varieties, it has previously been impossible to observe this across large segregating populations in field situations. The late planting, G2LA, further differs from the other two trials but, also being a stress trial (Supplemental Figure S1), is unsurprisingly more similar to the dryland planting, and the late flight dates are more important predictors for GY than early flight dates (Figure 3).

### 3.3 SNP–flight associations and functions of candidate genes

**FIGURE 5:**
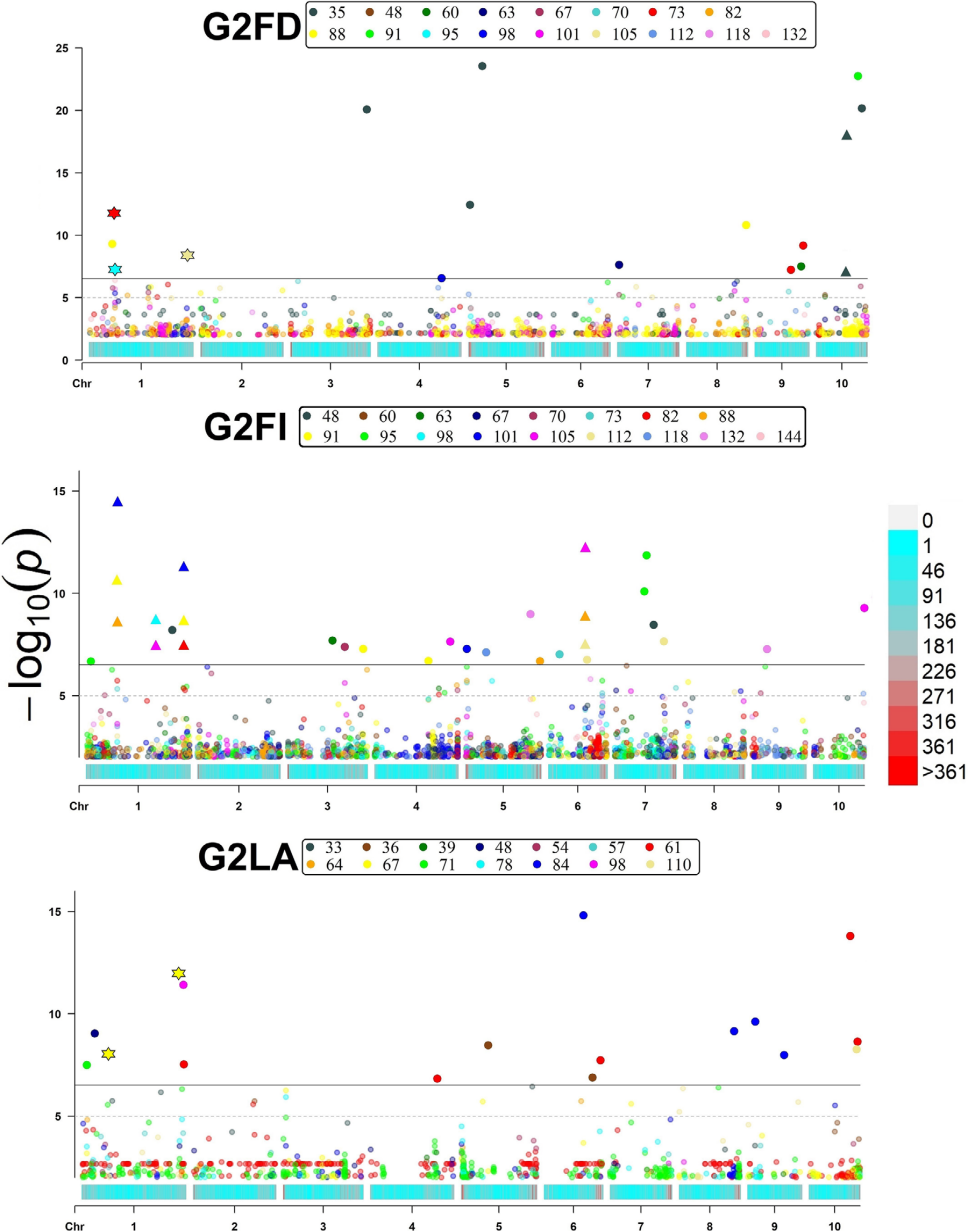
Combined Manhattan plots for plant heights of each flight using best linear unbiased predictions (BLUPs) of the mixed effects spatial model. The heat map at the bottom of each Manhattan plot shows the single nucleotide polymorphism (SNP) density (within 1 Mb window size) through the chromosomes. Scale of this heat map was given on the right side of Manhattan plot. Star shapes were used for colocalized SNPs detected in more than one trial. Triangle shapes were used for colocalized SNPs detected in more than one flight within any trial. Round shapes were used for unique SNPs. Each unique color within each trial represents the association between SNPs and plant heights of each flight; color charts are given inside the rectangles at the top of each Manhattan plots. G2FI, G2FD, and G2LA trial represent the optimal planting time with irrigation, optimal planting time without irrigation, and delayed planting time with irrigation, respectively

Over the three trials, the 99 percentile plant heights over 60 UAS flights, as well as GY, were genetically mapped using 153,252 SNP markers. Principal component analysis suggested that there were effectively three populations (Supplemental Figure S2). After accounting for population structure across all three trials, a total of 52 SNPs with *p* values above the Bonferroni correction were detected and annotated; seven were discovered to be common in more than one flight date, while 45 of these were flight‐time specific (Figure 5; Supplemental Table S1).

Of the seven SNPs that showed association with the trait in multiple flights and trials, three were located on chromosomes 1 and 10 and showed association in two flights, four were located on chromosomes 1 and 6 showed association across three flights (Supplemental Table S1). Additionally, two SNPs on chromosomes 1 showed association in two trials (Supplemental Table S1). Of the SNPs that showed association in two trials, none showed association in the same flight in different trials (Supplemental Table S1). In other words, the same SNPs were discovered in an earlier flight of the delayed planting trial (67 DAP in G2LA) but later flights in optimal planting (73 and 95 DAP in G2FD) (Supplemental Table S1); these were not the same calendar date flights. Discovering the common SNPs for plant heights belonging to different flights in more than one trial suggested that the same genetic cause of phenotypic variation in plant height can occur in different times under different management conditions such as delayed and optimal planting (Supplemental Table S1). This results in the discovery of the same loci at different time points of growth belonging to different management conditions.

**FIGURE 6:**
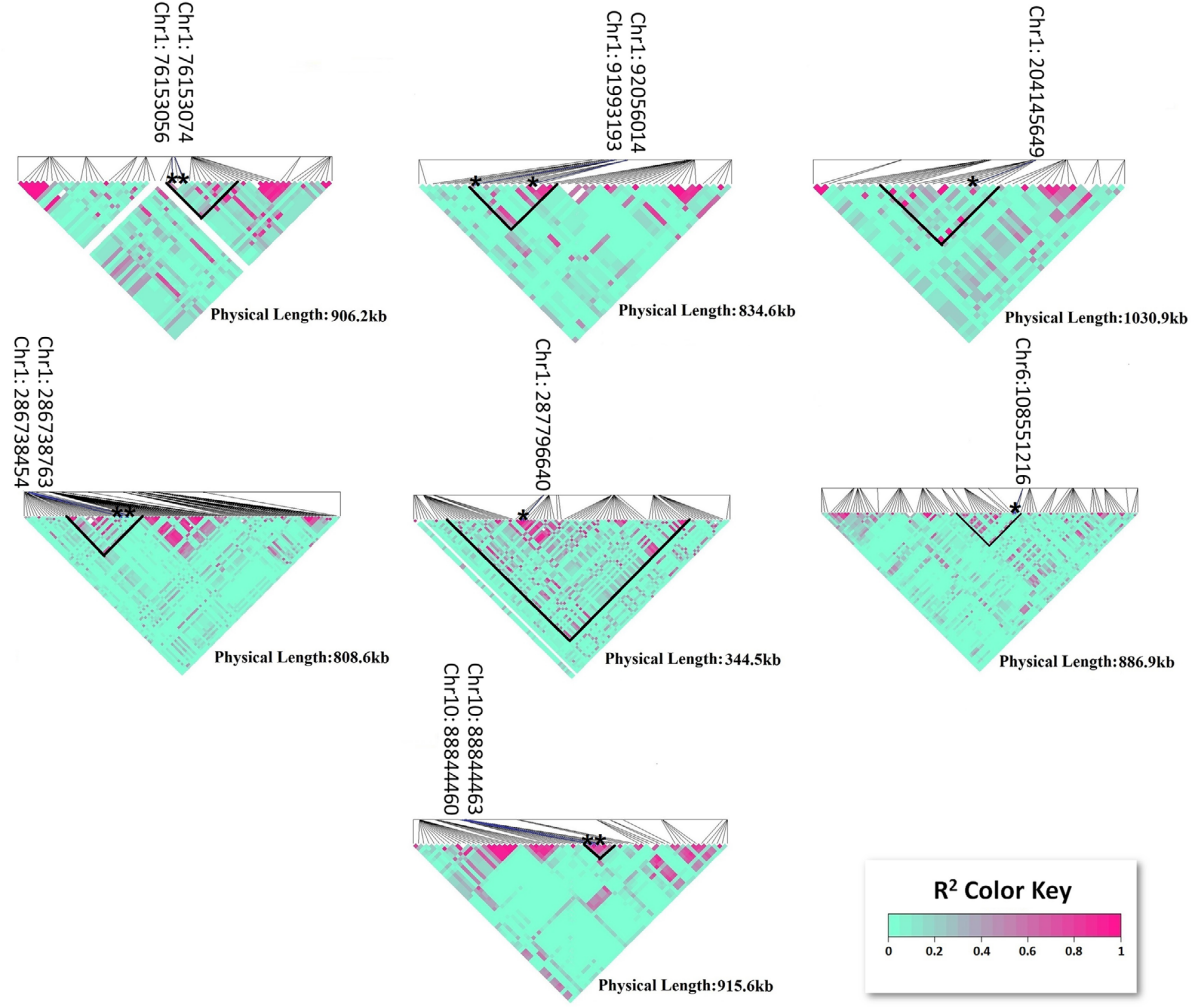
Linkage disequilibrium (LD) blocks of seven colocalized single nucleotide polymorphisms (SNPs) located in chromosomes 1, 6, and 10 associated with plant height. Black lines indicated the pairwise LD regions of the SNPs with 0.8 or higher *R* ^2^ within the physical lengths. Black stars showed the locations (base pair; bp) of the above SNPs

The ranges in LD observed in the regions surrounding the seven colocalized SNPs cannot exclude linked SNPs as causal (Figure 6). In chromosome 1, SNPs (76,153,074 and 76,153,056 bp) were found to be significant in flights of 73 and 95 DAP in G2FD and 67 DAP in G2LA, both were high stress trials. While these two SNPs discovered in G2FD 73 and 95 DAP flights with the highest logarithm of the odds scores (>6.55), these SNPs were also nearly significant later in 98, 112, 118, and 132 DAP flights (logarithm of the odds scores >5) in G2FD. The LD regions surrounding these two SNPs extends to 131 kbp (Chr1:76.08–76.21) (Figure 5) and contains four candidate genes in the LD interval. One of the candidate genes in LD interval is *GRMZM2G005630*, located 26 kbp away from these SNPs. *GRMZM2G005630* encodes the EID1‐like F‐box protein 2 that regulates ABA‐dependent signaling that manages seed germination, root growth, and transition to flowering time as well as the accumulation of anthocyanin under drought conditions in *Arabidopsis* (Koops et al., 2011). F‐box genes are one of the most diverse and largest gene families in higher plants; maize has ∼359 F‐box genes (Zhang et al., 2019; Jia et al., 2013). The F‐box protein family regulates plant growth and development as well as biotic and abiotic stresses in maize (Jia et al., 2013).

Two additional SNP associations were found close to each other on chromosome 1 (91,993,193 and 92,056,014 bp) and were significant at 88, 91, and 101 DAP in G2FI trials. The LD region is ∼66 kbp (Chr:91.99–92.06) (Figure 6) and contains three candidate genes. One of the candidate genes in LD is *GRMZM5G806839* that controls the AP2 transcription factor (also known as ereb44) in maize. *GRMZM5G806839* mediates response to multiple abiotic and biotic stresses (Kizis et al., 2001) such as southern corn leaf blight response (*Bipolaris maydis*) in maize (Bian et al., 2014).

Another SNP association on chromosome 1 (204,145,649 bp) was found at 98 and 105 DAP in G2FI. The surrounding LD region of this SNP is ∼170 kbp (Chr1:203.98–204.15) and contains seven candidate genes in the interval, of which none have been previously identified. One of the candidate genes, *GRMZM2G016210*, contains this SNP in its genic region and includes the umc1122 simple sequence repeat marker. The flanking region between umc1035 and umc1122 markers was previously found to control plant height at different growth stages with different effect sizes in maize (Yan et al., 2003). *Qph1*, which is a rare SNP in *Bractic2* gene, is one of the well‐known QTL controlling plant height that has also been mapped near umc1122 (Xing et al., 2015). This QTL is congruent with the idea of a major QTL often described based on studies largely conducted in optimal environmental conditions of the U.S. Midwest and Europe would only be detected in Texas under the most favorably managed trial.

Two chromosome 1 SNP associations (286,738,454 and 286,738,763 bp) were found at 82, 91, and 101 DAP in G2FI. The LD block around the SNPs is ∼66 kbp (Chr1:286.73–286.79) (Figure 6) and it contains three candidate genes. *GRMZM2G028286* is one of the candidate genes in the LD region and it is reported to control the cellulose biosynthesis pathway (Kianifariz, 2017). In a previous study, *GRMZM2G028286* was found to be downregulated when the *NUT1* mutant gene is active; the *NUT1* mutant gene causes an erratic tassel phenotype (e.g. tassel browning and sterility) especially under drought stress because of a defect in water transport (Dong et al., 2020) as well as reduced plant height in maize (Dong et al., 2020). Another candidate gene in this LD region is *GRMZM2G028151* (ereb184‐AP2‐EREBP transcription factor) that is an AP2‐like ethylene responsive transcription factor and it was reported to be upregulated under heat stress (Casaretto et al., 2016).

The chromosome 1 SNP association (287,796,640 bp) was found at 67 and 105 DAP in G2LA and G2FD trials. The LD region surrounding this SNP is ∼253 kbp (Chr1:287.66–287.92) and contains 11 candidate genes. One of the candidate genes within the LD interval is *GRMZM2G122139*, and this SNP is also found in its genic region. This candidate gene encodes Cytosolic purine 5‐nucleotidase. This enzyme was purified from maize microsomes (Carter & Tipton, 1985) and wheat seedling leaves and potato (*Solanum tuberosum* L.) (Polya, 1974, 1975), suggesting a relationship with a cyclic nucleotide regulatory system in higher plants. Another candidate gene, *GRMZM2G072806*, encodes the ubiquinone oxidoreductase enzyme; its activity plays a role on NAD(H) biosynthesis by transporting a hydride ion (H^−^). This gene could disrupt regular enzymatic activities causing oxidative stress (Moller, 2001).

A SNP association on chromosome 6 (108,551,216 bp) was found at 88, 105, and 112 DAP in the G2FI trial. The LD region around this SNP is ∼180 kbp (Chr6:108.40–108.58) (Figure 6) and contains nine genes. One candidate gene in this interval is *Zm00001d03699*4. This candidate gene has been reported to encode trichome birefringence‐like proteins that were localized in Golgi and transfers the acetyl groups to xylan resulting in the elongation of the xylan (Xiong et al., 2013). Two trichome birefringence‐like‐related recessive mutants in rice were found to reduce the xylan monoacetylation in cell wall and cause the reduced plant height and susceptibility to leaf blight (Gao et al., 2017).

Chromosome 10 SNP associations (88,841,430 and 88,845,809 bp) were found at 35 DAP in the G2FD trial. The LD region around these SNPs is ∼6 kbp (Chr10: 88.84–88.85) (Figure 6) and contains two candidate genes. One of the candidate genes in this interval, *GRMZM2G089484* (MAP kinase), was reported to be a possible candidate associated with northern corn leaf blight resistance caused by *Exserohilum turcicum* (Ding et al., 2015). Another candidate gene, *GRMZM2G422090* (GTP‐binding protein), is related to the control of cell elongation resulting from the stimulation of the auxin hormone (Terryn et al., 1993).

**FIGURE 7:**
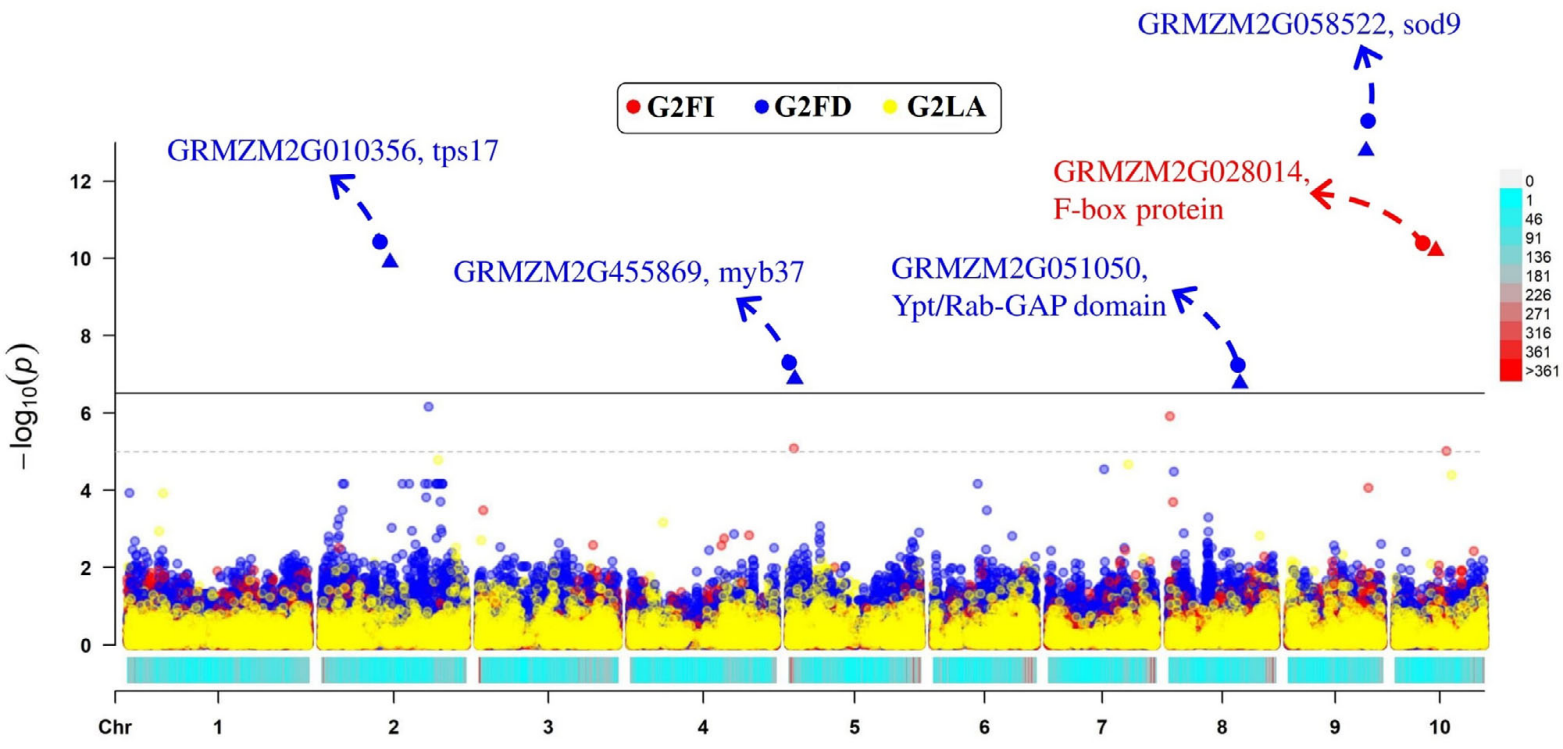
Single nucleotide polymorphisms (SNPs) associated with hybrid grain yield (t ha^−1^) for G2FI, G2FD, and G2LA trials. G2FI, G2FD, and G2LA trial represent the optimal planting time with irrigation, optimal planting time without irrigation (dryland), and delayed planting time with irrigation respectively. The heat map at the bottom of the Manhattan plot shows the SNP density (within 1 Mb window size) through the chromosomes. Scale of this heat map was given on the right side of Manhattan plot

Five SNPs that are in the genic region of *GRMZM2G010356*, *GRMZM2G455869*, *GRMZM2G051050*, *GRMZM2G058522*, and *GRMZM2G028014* were discovered for GY in G2FD and in G2FI (Figure 7). *GRMZM2G010356*, also known as *tps17*, involves the terpene synthase biosynthesis that contributes the defense mechanisms against parasitoids and pathogens in maize (Block et al., 2019 ; Liang et al., 2018). *GRMZM2G455869*, also known as *MYB37*, regulates the elongation of bundle sheath cells in maize (Chang et al., 2012). *GRMZM2G051050*, also known as *Ypt/Rab* domain of the gyp1p superfamily protein, was found to be related with yield under high infestation of Mediterranean corn borer (*Sesamia nonagrioides* Lefebvere) in maize (Jiménez‐Galindo et al., 2018). *GRMZM2G058522*, also known as superoxide dismutase, creates an initial defense response against reactive oxygen species that stemmed from exposure of numerous environmental stresses such as drought, intense UV lights, air pollutants, and chilling temperatures (Alscher, Erturk & Heath, 2002). GRMZM2G028014 is one of member in F‐box protein family in maize that is responsible for regulation of protein degradation, signal perception and transduction pathways inside and often outside of cells regions in maize (Jia, Wu, Li, Huang & Zheng, 2013)

### 3.4 Contributions of UAS to association studies

High‐throughput phenotyping by UAS captured phenotypic variation arising from the response of diverse hybrids to various abiotic stresses across plant development stages. When this variation was combined with genomic information in an association mapping study, SNPs were detected and linked candidate genes were identified for plant heights of different flights and GY involved in coping with many environmental stress factors (Figures 5 and 7; Supplemental Table S1). The resulting candidate gene annotations for these SNPs showed that segregating loci controlling numerous biological functions orchestrate differences between hybrids temporally throughout plant development, yet there were substantial differences under the two abiotic stresses. Some of these loci conditioned variation across multiple time periods, while others appeared at a specific time point and under specific growing conditions. Plant height is a result of many environmental stress factors during the development periods, causing temporal modifications in plant height depending on different growth stages. The temporal variations in plant height can therefore be evaluated as a phenotypic consequence of the response of maize hybrids to environmental stresses during growth. When temporal phenotypic data collected by UAS are combined with genomic (e.g. GBS) data, phenotypic plasticity of plant height occurred within growth stages and underlying genes can be scrutinized with higher accuracy and resolution.

### 3.5 Cumulative SNP effects

**FIGURE 8:**
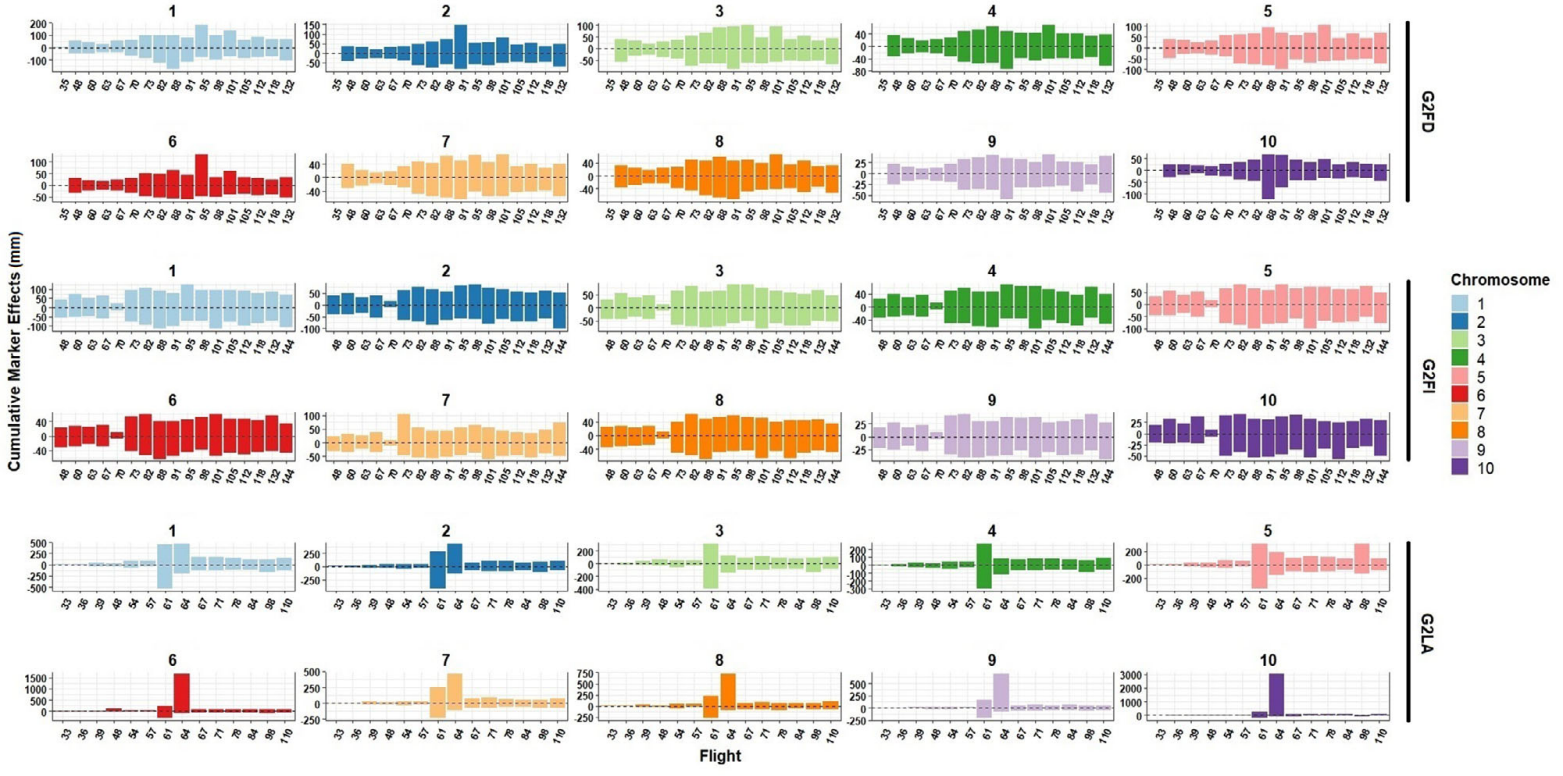
Temporal cumulative marker effects of each chromosome (negative and positive) for each flight of each trial. The cumulative effects of whole‐genome‐wide markers (*y* axis, mm) were dependent upon the flights (*x* axis, DAP). G2FI, G2FD, and G2LA trial represent the optimal planting time with irrigation, optimal planting time without irrigation, and delayed planting time with irrigation, respectively

Genomic prediction and selection approaches rely on contributions of genome‐wide genetic markers. However, the phenotypes used in these studies typically have been low dimensional and low throughput obtained from limited time intervals (one or a few times) or small sample sizes. Therefore, different contributions of each locus at different time points for the same phenotype of interest will remain unobserved, with the exception of those effects that make substantial enough contribution to be detected during the terminal measurements. As we were able to only obtain genotypes of 158 hybrids of the over 270 hybrids studies, this limited these studies potential detection ability. In this study, cumulative SNP marker effects of each chromosome were found to differ depending on the flight date (Figure 8). However, like repeatability (Figure 2) the cumulative marker effects likely varied over time because of both unexplained error and different sources of phenotypic variation at different flights. For instance, there is a peak in cumulative marker effects at 64 DAP in the G2LA trial (Figure 8). The 64 DAP also had the highest repeatability value in G2LA trial (Figure 2). This is a particular example where repeatability and total cumulative marker effects were highest in G2LA trial, which may help explain that variation by pedigree at 64 DAP in G2LA trial has stemmed mostly from marker effects contributed by many regions across the genome (Figure 8). Furthermore, the different results we observed between the three management conditions demonstrated that G×E interplay, illustrated here by the interaction of hybrids and trials, had a cryptic influence on phenotypic variation (Gage et al., 2017). Plasticity is occurring even within an environment temporally as demonstrated by fluctuating marker effects, repeatability values, and explained variation by pedigree. The flights revealed this temporal within environmental variation, which was combined with G×E interaction to better scrutinize phenotypic plasticity. This study was only carried out within 1 yr, but additional years would likely further increase the amount of G×E interaction effects found.

### 3.6 Temporal genomic predictions

**FIGURE 9:**
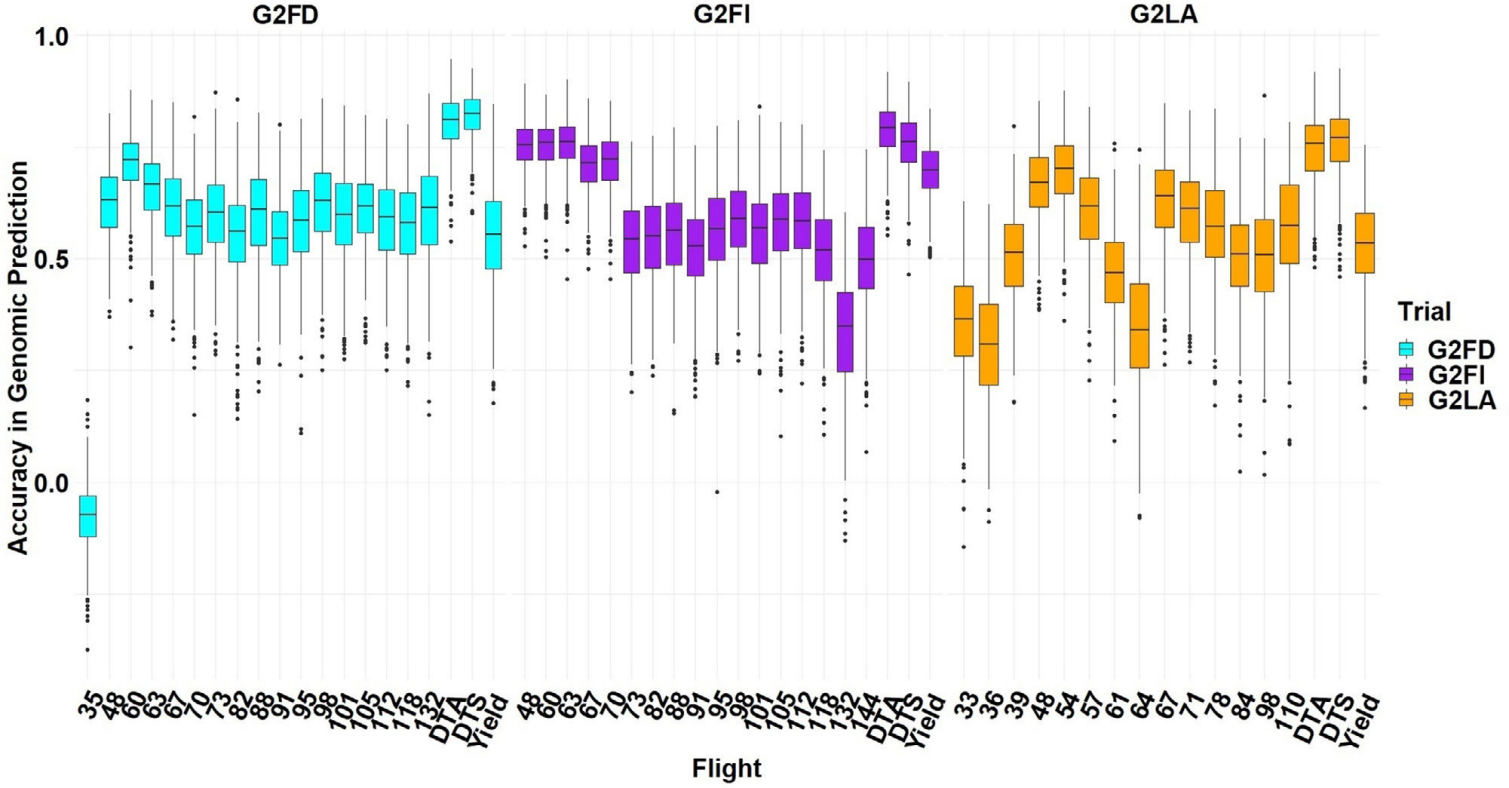
Genomic prediction accuracies (*r*_gpa_) (*y* axes) for flights (*x* axis as days after planting [DAP]) as well as for days to anthesis (DTA), days to silking (DTS), and yield in three trials. G2FI, G2FD, and G2LA trial represent the optimal planting time with irrigation, optimal planting time without irrigation, and delayed planting time with irrigation respectively

Genomic prediction accuracies for plant heights belonging to each flight in each trial were illustrated in Figure 9. Genomic prediction accuracies for DTA, DTS, and GY were also calculated for each trial (Figure 9).

Our findings showed *r*_gpa_ for plant heights captured by flights fluctuated within G2FD (−0.06∼0.73), G2FI (0.33∼0.76), and G2LA (0.26∼0.78) trials (Figure 9). Genomic prediction accuracies for 40−60 DAP flights (preflowering) were surprisingly higher than in later flights for each trial (Figure 9). Dynamic patterns of *r*_gpa_ tended to decrease toward later flights, especially in the G2FI optimal trial. In other words, flights before initiation of flowering times had higher *r*_gpa_ as opposed to later flights in each trial (Figure 9). In the findings of this study, the time‐dependent alteration of genomic prediction results (Figure 9) and the genome‐wide marker effects (Figure 8) revealed that the same markers (loci) may have diverse effects on plant height depending on different growth periods in maize.

## 4 CONCLUSION

The estimations of breeding values for genetic materials using HTP combined in association with high‐throughput genotyping data (e.g., GBS) has only begun to be explored. This study was among the first evaluating a high number of hybrid maize plots with UAS in a genome‐wide association study to discover loci (and where LD permitted, candidate genes) and the prediction ability for the phenotype of interest. Within the scope of this study, early growth stages were found to have undiscovered phenotypic variation controlled by unique loci not found at later stages; this is important to increase our understanding of complex traits in crops. Major conclusions of this study were that (a) earlier plant height data had more importance for GY under optimal planting and irrigation, whereas later plant height data had more importance in terms of yield under stressed conditions; (b) genomic prediction accuracies (*r*_gpa_) varied for plant heights belonging to different growth stages; and (c) a large number of plant height data captured by UAS can be used as predictors to predict yielding hybrids via RF algorithm.

## CONFLICT OF INTEREST STATEMENT

Authors declared that there is no conflict of interest.

## AUTHOR CONTRIBUTIONS

A.A.: Original draft, formal analysis, data curation, bioinformatic analysis, methodology, data extraction from orthomosaics, conceptualization, investigation, field observation, review and editing (lead); S.C.M.: conceptualization, funding acquisition, methodology, project administration, supervision, resources, review and editing (lead and supporting); S.L.A: formal analysis, data curation, data extraction from orthomosaics, conceptualization, investigation, field observation, review and editing (supporting); S.C.P.: conceptualization, funding acquisition, resources, review and editing (supporting), software, supervision; L. M.: conceptualization, data curation, formal analysis, investigation, methodology, review and editing (supporting); M.C.R.: bioinformatic, conceptualization, review and editing (supporting); N.D.L: Genome to field project (G2F) coordination, bioinformatic, conceptualization, investigation, project administration, review and editing (supporting).

## Supporting information

Supplemental Table S1. contains the discovered SNPs (with p‐values higher than FDR and Bonferonni threshold) in Manhattan plots in Figure 5 with their SNP names, chromosomes, physical positions (bp), p values, p values as ‐log100, minor allele frequencies (MAF), SNP effects, explained percent variances, flight times as DAP, trials, spanning regions, candidate genes and descriptions information respectively.

Supplemental Figure S1. Differences in growing degree day accumulation and photoperiod in the three trials.

Supplemental Figure S2. Population structure of hybrids using 153,252 filtered SNPs belonging to the 158 hybrids with genotype information.

Supplemental Figure S3. Pearson correlation coefficients among the predicted plant heights (UAS_PEBVs_) belonging to different flights of each trial.
